# Supplementary material for: Impact of health systems interventions in primary health settings on type 2 diabetes care and health outcomes among adults in West Africa: A systematic review protocol
Source: PLoS One. 2024 Nov 8;19(11):e0291474. doi: 10.1371/journal.pone.0291474 (PMC11548752; doi:10.1371/journal.pone.0291474)
Supplement: S1 File — (PDF) [file pone.0291474.s001.pdf]

S1 File. Search strategy as at February 7, 2024

Database	Search Query	Total number of resulting articles
PubMed	("Health Services"[MeSH Terms] OR "Primary Health Care"[MeSH Terms] OR "Delivery of Health Care"[MeSH Terms] OR "Health Facilities"[MeSH Terms] OR "health care facilities, manpower, and services"[MeSH Terms] OR "Healthcare Financing"[MeSH Terms] OR "insurance, health, reimbursement"[MeSH Terms] OR "Health Information Systems"[MeSH Terms] OR "Equipment and Supplies"[MeSH Terms] OR "health facilit*"[Text Word] OR "primary healthcare"[Text Word] OR "Health Services"[Text Word] OR "healthcare delivery"[Text Word] OR "delivery of healthcare"[Text Word]) AND ("diabetes mellitus, type 2"[MeSH Terms] OR "Glycemic Control"[MeSH Terms] OR "Diabetes Complications"[MeSH Terms] OR "Hyperglycemia"[MeSH Terms] OR "type 2 diabet*"[Text Word] OR "Glycemic Control"[Text Word] OR "Hyperglycemia"[Text Word]) AND ("africa, western"[MeSH Terms] OR "Benin"[Text Word] OR "Burkina Faso"[Text Word] OR "Cabo Verde"[Text Word] OR "Cote d'ivoire"[Text Word] OR "Gambia"[Text Word] OR "GHANA"[Text Word] OR "Guinea"[Text Word] OR "Guinea-Bissau"[Text Word] OR "Liberia"[Text Word] OR "Mali"[Text Word] OR "Mauritania"[Text Word] OR "Niger"[Text Word] OR "Nigeria"[Text Word] OR "Senegal"[Text Word] OR "Sierra Leone"[Text Word] OR "Togo"[Text Word])) AND (1999:2023[pdat])	343
Google Scholar	("health facility" OR "primary health care" OR "health services" OR "health care delivery" OR "health system intervention") AND ("type 2 diabetes" OR "glycemic control" OR "hyperglycemia") AND "West Africa" Diabetes "RCT" -pregnancy, -gestational<br><br>("health facility" OR "primary health care" OR "health services" OR "health care delivery" OR "health system intervention") AND ("type 2 diabetes" OR "glycemic control" OR "hyperglycemia") AND "West Africa" Diabetes "CCT" -pregnancy, -gestational<br><br>("health facility" OR "primary health care" OR "health services" OR "health care delivery" OR "health system intervention") AND ("type 2 diabetes" OR "glycemic control" OR "hyperglycemia") AND "West Africa" Diabetes "quasi experiment" -pregnancy, -gestational<br><br>("health facility" OR "primary health care" OR "health services" OR "health care delivery" OR "health system intervention") AND ("type 2 diabetes" OR "glycemic control" OR "hyperglycemia") AND "West Africa" Diabetes "clinical trial" -pregnancy, -gestational	57<br><br>18<br><br>4<br><br>251

CINAHL	health facility OR primary healthcare OR health services OR delivery of health care OR Health Care Facilities, Manpower, and Services OR healthcare financing OR Insurance, Health, Reimbursement OR health information systems OR equipment and supplies AND type 2 diabetes OR glycemic control OR hyperglycemia OR diabetes mellitus type 2 OR diabetes complications OR diabetes adherence AND Benin OR burkina faso OR cabo verde OR cote d'ivoire OR gambia OR ghana OR guinea OR guinea bissau OR liberia OR mali OR mauritania OR niger OR nigeria OR senegal OR sierra leone OR togo AND rct or randomised control trial OR clinical controlled trial	328
Cairn. Info	1. 'accès' ET 'diabètes' ET 'résultats' ET 'afrique de l'ouest'<br>2. interventions pour le diabète du type 2 en Afrique de l'ouest<br>3. interventions pour les diabetes en Afrique de l’ouest<br>4. diabète de type ii en afrique de l'ouest<br>5. diabète ET 'West Africa'<br>6. ‘diabetes’ and ‘africa’<br>7. 'diabetes' AND 'west africa'<br>8. 'type 2 diabetes' and 'west africa'<br>9. 'interventions' and 'type 2 diabetes' and 'west africa'<br>10. 'interventions' and 'type 2 diabetes'<br>11. 'interventions' and 'type 2 diabetes' and 'outcomes'<br><br>1 OR 2 OR 3 OR 4 OR 5 OR 6 OR 7 OR 8 OR 9 OR 10 OR 11	23
