# Supplementary figures and images for: Impact of health systems interventions in primary health settings on type 2 diabetes care and health outcomes among adults in West Africa: A systematic review protocol
Source: PLoS One. 2024 Nov 8;19(11):e0291474. doi: 10.1371/journal.pone.0291474 (PMC11548752; doi:10.1371/journal.pone.0291474)

## S2 File. Study selection flowchart.

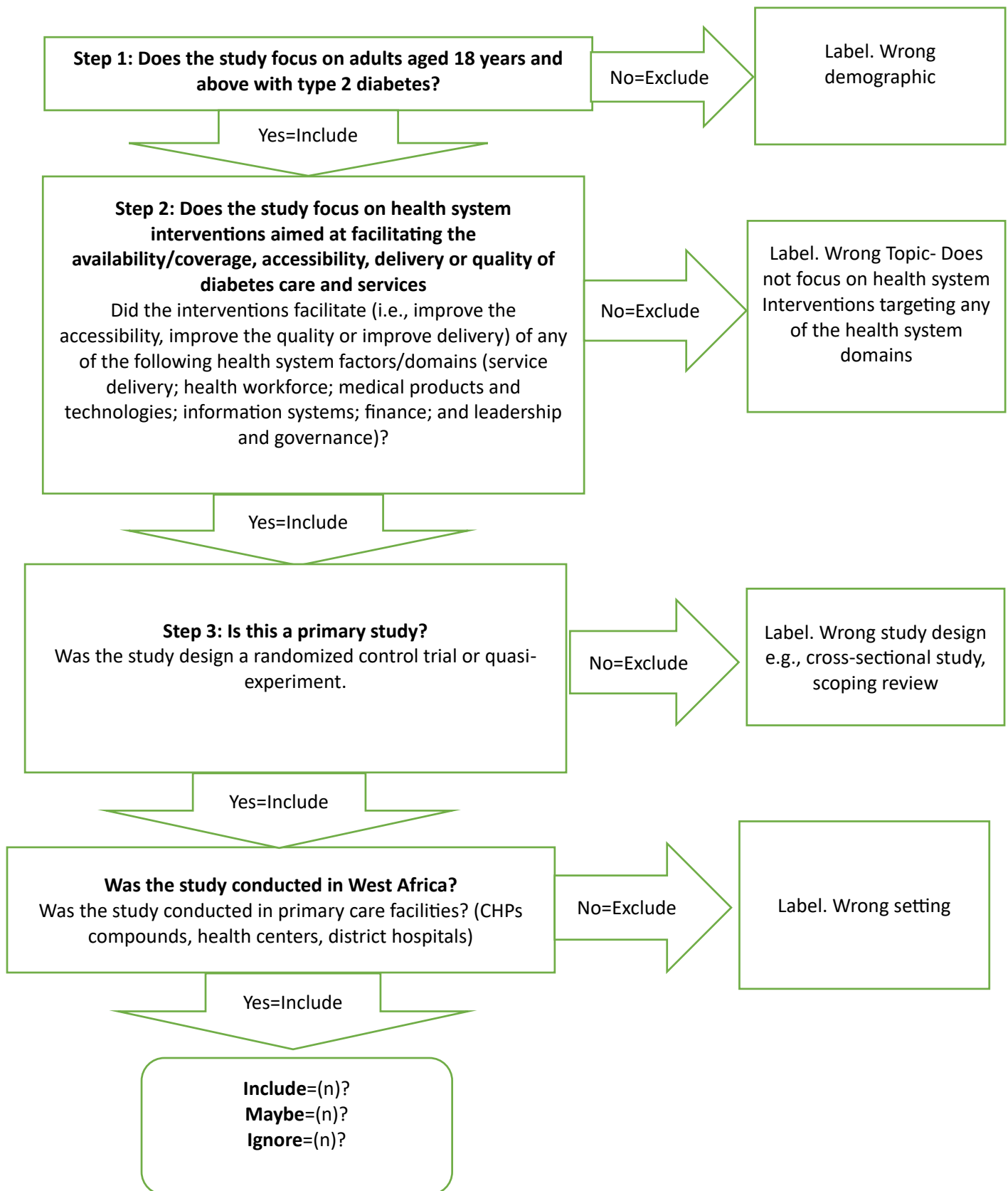

Supplement: S2 File — (PDF) [file pone.0291474.s002.pdf]
