# Supplementary material for: Impact of health systems interventions in primary health settings on type 2 diabetes care and health outcomes among adults in West Africa: A systematic review protocol
Source: PLoS One. 2024 Nov 8;19(11):e0291474. doi: 10.1371/journal.pone.0291474 (PMC11548752; doi:10.1371/journal.pone.0291474)
Supplement: S1 Table — (DOCX) [file pone.0291474.s004.docx]

**S1 Table. Data extraction form**

| First Author (year of publication) | Country | Study design | Sample Size | Intervention | Sample population | Study site (e.g., CHPs, district hospital, sub-regional hospital) | Intervention described | Duration of Intervention | Control | Health system domain explored | | | | outcomes | | |
| --- | --- | --- | --- | --- | --- | --- | --- | --- | --- | --- | --- | --- | --- | --- | --- | --- |
|  |  |  |  |  |  |  |  |  |  | Service delivery | Health Workforce | Supply chain | Leadership and governance | Glycemic control | Awareness | Associated outcomes |
