# Supplementary material for: Impact of health systems interventions in primary health settings on type 2 diabetes care and health outcomes among adults in West Africa: A systematic review protocol
Source: PLoS One. 2024 Nov 8;19(11):e0291474. doi: 10.1371/journal.pone.0291474 (PMC11548752; doi:10.1371/journal.pone.0291474)
Supplement: Attachment — Submitted filename: Response to Reviewers.docx [file pone.0291474.s005.docx]

Eugene Paa Kofi Bondzie

Ghana College of Physicians and Surgeons

Accra, Ghana

[eugenebondzie@gmail.com](mailto:eugenebondzie@gmail.com)

+233592182279

13^th^ February, 2024

Hubert Amu

Academic Editor

PLOS ONE

Dear Editor,

Thank you for considering our manuscript “Impact of health system interventions in primary health settings on type 2 diabetes care and health outcomes among adults in West Africa: a systematic review protocol” for publication in your esteemed journal. We appreciate the time and effort devoted by the editors and reviewers to providing feedback on our work.

We have carefully considered the comments and revised the manuscript accordingly.

Our responses to the editor and reviewer’s comments have been outlined below. The comments are in ***bold-italic font*** and our responses are in regular font.

We believe these revisions have helped improve the quality and clarity of our manuscript significantly.

Your efforts in handling the manuscript are greatly appreciated.

Yours Sincerely,

Eugene Paa Kofi Bondzie.

(On behalf of all authors)

**EDITOR COMMENTS AND RESPONSES**

***1. Please ensure that your manuscript meets PLOS ONE’S style Requirements, including those for file naming****.*

Response:

Thank you. We followed the PLOS ONE style templates to write the manuscript and organized file naming. We uploaded all figures separately.

***2. Please note that funding information should not appear in any section or other areas of your manuscript. We will only publish funding information present in the Funding Statement section of the online submission form. Please remove any funding-related text from the manuscript.***

Response:

The funding statement has been removed from the manuscript.

***3. Please review your reference list to ensure that it is complete and correct. If you have cited papers that have been retracted, please include the rationale for doing so in the manuscript text, or remove these references and replace them with relevant current references. Any changes to the reference list should be mentioned in the rebuttal letter that accompanies your revised manuscript. If you need to cite a retracted article, indicate the article’s retracted status in the References list and also include a citation and full reference for the retraction notice.***

Response:

We have made changes to reference number 6 on line 391-392. Reference number 6 currently states as:

IDF_Atlas_10th_Edition_2021.pdf [Internet]. [cited 2023 Jun 19]. Available from: <https://diabetesatlas.org/idfawp/resource-files/2021/07/IDF_Atlas_10th_Edition_2021.pdf>

We also provided additional references in response to **reviewer #1’s** comment on the Background information. Reference numbers 9-14 and 23-31

Line 396-411

9.	International Diabetes Federation [Internet]. [cited 2024 Feb 2]. Nigeria. Available from: https://idf.org/our-network/regions-and-members/africa/members/nigeria/

10.	International Diabetes Federation [Internet]. [cited 2024 Feb 2]. Ghana. Available from: https://idf.org/our-network/regions-and-members/africa/members/ghana/

11.	International Diabetes Federation [Internet]. [cited 2024 Feb 2]. Burkina Faso. Available from: https://idf.org/our-network/regions-and-members/africa/members/burkina-faso/

12.	Ibrahim AO, Agboola SM, Elegbede OT, Ismail WO, Agbesanwa TA, Omolayo TA. Glycemic control and its association with sociodemographics, comorbid conditions, and medication adherence among patients with type 2 diabetes in southwestern Nigeria. J Int Med Res. 2021 Oct;49(10):3000605211044040.

13.	Cedrick LM, Lubaki JPF, Francois LB, Gboyega OA, Philippe LN. Prevalence and determinants of poor glycaemic control amongst patients with diabetes followed at Vanga Evangelical Hospital, Democratic Republic of the Congo. Afr J Prim Health Care Fam Med. 2021 Apr 30;13(1):2664.

14.	Camara A, Baldé NM, Sobngwi-Tambekou J, Kengne AP, Diallo MM, Tchatchoua APK, et al. Poor glycemic control in type 2 diabetes in the South of the Sahara: the issue of limited access to an HbA1c test. Diabetes Res Clin Pract. 2015 Apr;108(1):187–92

Line 432-460

23.	de-Graft Aikins A, Kushitor M, Koram K, Gyamfi S, Ogedegbe G. Chronic non-communicable diseases and the challenge of universal health coverage: insights from community-based cardiovascular disease research in urban poor communities in Accra, Ghana. BMC Public Health. 2014 Jun;14(S2):S3.

24.	Kushitor MK, Boatemaa S. The double burden of disease and the challenge of health access: Evidence from Access, Bottlenecks, Cost and Equity facility survey in Ghana. PLOS ONE. 2018 Mar 23;13(3):e0194677.

25.	Bosu WK. A Comprehensive Review of the Policy and Programmatic Response to Chronic Non-Communicable Disease in Ghana. Ghana Med J. 2012 Jun;46(2 Suppl):69–78.

26.	Ajisegiri WS, Abimbola S, Tesema AG, Odusanya OO, Ojji DB, Peiris D, et al. Aligning policymaking in decentralized health systems: Evaluation of strategies to prevent and control non-communicable diseases in Nigeria. PLOS Glob Public Health. 2021 Nov 10;1(11):e0000050.

27.	Ouedraogo M, Sanou D, Ouedraogo O, Zongo U, Hama-Ba F, Savadogo A. An overview of governance of noncommunicable diseases (NCDs) in West African countries. Pan Afr Med J [Internet]. 2023 Apr 26 [cited 2024 Feb 7];44(203). Available from: https://www.panafrican-med-journal.com/content/article/44/203/full

28.	Tesema AG, Ajisegiri WS, Abimbola S, Balane C, Kengne AP, Shiferaw F, et al. How well are non-communicable disease services being integrated into primary health care in Africa: A review of progress against World Health Organization’s African regional targets. PLOS ONE. 2020 Oct 22;15(10):e0240984.

29.	Cissé K, Kouanda S, Coppieters’t Wallant Y, Kirakoya-Samadoulougou F. Awareness, Treatment, and Control of Hypertension among the Adult Population in Burkina Faso: Evidence from a Nationwide Population-Based Survey. Int J Hypertens. 2021 Sep 29;2021:5547661.

30.	Ateudjieu J, Muhamed Awolu M, Yakum M, Bayiha EJ, AchombwomThomas V, Watcho P, et al. Assessing The Availability And Readiness Of Diabetes Healthcare Service In The West Region Of Cameroon. Int J Adv Res Publ. 2018 Feb 26;2:79–84.

31.	Akinwumi AF, Esimai OA, Arije O, Ojo TO, Esan OT. Preparedness of primary health care facilities on implementation of essential non-communicable disease interventions in Osun State South-West Nigeria: a rural–urban comparative study. BMC Health Serv Res. 2023 Feb 14;23(1):154.

***4. Have the authors described where all data underlying the findings will be made available when the study is complete?***

***Reviewer #1: No***

***Reviewer #2: Yes***

Response:

We have added the following lines to clarify this question:

Line 354-356

All results will be made fully available upon completion of this study.

**REVIEWERS’ COMMENTS AND RESPONSES**

**Reviewer #1:**

**“*Authors Bondzie et al, have written a nicely crafted protocol for a SR on an important topic. Congratulations. Below are some observations which I feel would help the protocol to improve in what it intends to serve.***

***Title: I would rather choose effectiveness over impact in the title”.***

Response:

Thank you for the suggestion. We opted for impact over effectiveness in the title because whereas effectiveness often refers to the consequence of an action and concerned with the most closely attributable results, impact on the other hand implies the influence of an action on broader changes and effects. E.g. Effectiveness of Metformin on blood glucose explores the direct consequence of metformin on blood glucose. Our study seeks to identify the influence of health system interventions on both intrinsic, and final healthcare outcomes for type 2 diabetes. Also, acknowledging that biomedical interventions and life style interventions have influence on disease outcomes.

***“Financial Disclosure: Researchers mention that this research was conducted …… While this is just a protocol for a SR, is that correct to mention to write that way. Is this SR funded with that grant, is this SR a part of another big study or some project. Please revisit and mention it clearly what exactly it is and mention it in the right way.”***

Response:

Thank you for your comment. We have removed the financial disclosure from the manuscript per the advice of the editor. However, this systematic review will be conducted as part of the National Institute of Health and Research’s West African project on Non-Communicable diseases. The funding statement has been corrected on the online submission form.

***“Background:***

***Please check referencing. Ref no 6 for e.g., has been provided with an inappropriate way in the references, please check the authors you have written for this reference and correct it.”***

Response:

Thank you for pointing this out. Reference no. 6 has been corrected on line 413-415

IDF_Atlas_10th_Edition_2021.pdf [Internet]. [cited 2023 Jun 19]. Available from: [https://diabetesatlas.org/idfawp/resource-files/2021/07/IDF_Atlas_10th_Edition_2021.pdf”-](https://diabetesatlas.org/idfawp/resource-files/2021/07/IDF_Atlas_10th_Edition_2021.pdf)

***“Adding the burden of disease information of Type 2 DM would strengthen the background, rather than just the proportion of people with DM in the region. Also, you could narrow down the information to West Africa where you plan to focus your SR on.***

***Till line 107, the authors talk about sub–Saharan Africa without any mention of west Africa, and then in the next paragraph line 108 – 114, the authors suddenly mention the need of the study in West Africa. We do not find a clear transition to this para. In addition, the authors have probably failed to illustrate the current health system situation. A clear and coherent information about the disease burden and health care delivery system situation including availability and readiness with a focus on west African region would set a clear background information for the proposed SR.”***

Response:

Thank you for your suggestion. We have added the following lines to highlight the burden of type 2 diabetes in West Africa:

Line 79-81

“West African countries have estimated prevalence rates of 3.7% in Nigeria, 3.95% in Ghana and 1.7% in Burkina Faso. Despite these numbers, glycemic control remains suboptimal”

We also added the following lines to highlight the health care delivery system situation for Non-communicable diseases in West Africa:

Line 93-100

“The primary healthcare system in West Africa is rather oriented towards the management of communicable diseases. Whereas, NCD management appears to be less effective. Healthcare policies and intervention programs mainly exist on paper in many parts of the region. Primary health care facilities are ill prepared to implement essential interventions for type 2 diabetes control. Results from assessment surveys indicate lack of essential medicines, basic equipment and diagnostics; lack of qualified trained personnel; and poor referral systems for the management of diabetes.”

***“Research Questions:
I would rephrase the research question as below:
How do health systems interventions influence healthcare in primary care settings and health outcomes of type 2 diabetes among adults in West Africa?”***

Response:

Thank you very much for your suggestion. We have rephrased our research question to:

“How do health systems interventions influence healthcare in primary care settings and health outcomes of type 2 diabetes among adults in West Africa?” - Line 146-147

***“Objectives:
The way the objectives are written, it looks like general and specific objectives appear to be written as if they are objective 1, 2 & 3. Please have a look once again. Provide a more general/overall objective in general objective, if you would like to follow this structure of general and specific objectives. As per your research question and topic of SR, current general objective cannot be your general objective. It just says to identify the interventions…… is that what your SR is all about?
Please reorganize the objectives section. Furthermore, in the last objective you mention associated health outcomes but while trying to qualify it as example you mention service availability, service utilization….. do you call these as outcomes??”***

Response:

Thank you for this thoughtful comment. We have re-organized the objectives. They have been written as objective 1, 2 & 3. We have also re-worded the associated outcomes in the last objective

Line 149-155

1. To identify the health system interventions in primary health settings that influence the, accessibility, delivery and quality of type 2 diabetes care among adults in West Africa.

2. To explore the evidence on the effectiveness of these system-level interventions on glycemic control, awareness and treatment adherence.

3. To explore the impact of these system-level interventions on any associated health outcomes (i.e. any other patient outcomes or facility level outcomes associated with an intervention).

***“Minor comments on methods:
While describing the PICOS, I would follow the order as in the table.”***

Thank you for your suggestion.

We have rearranged the description of the PICOS to align with the order in Fig 2.

**Reviewer #2:**

**“suggest to add one more review question: Does an improvement on health outcomes could be observed among the population with various socioeconomic disparities?”**

Response:

Thank you for your thoughtful suggestion. We have considered this particular objective. However, while we recognize the potential value of an additional question, we would like to maintain the original focus of the review as per the registered protocol.
