# Supplementary material for: Impact of health systems interventions in primary health settings on type 2 diabetes care and health outcomes among adults in West Africa: A systematic review protocol
Source: PLoS One. 2024 Nov 8;19(11):e0291474. doi: 10.1371/journal.pone.0291474 (PMC11548752; doi:10.1371/journal.pone.0291474)
Supplement: Attachment — Submitted filename: Response to Reviewers.docx [file pone.0291474.s006.docx]

Eugene Paa Kofi Bondzie

Ghana College of Physicians and Surgeons

Accra, Ghana

[eugenebondzie@gmail.com](mailto:eugenebondzie@gmail.com)

+233592182279

9^th^ June, 2024

Blessing Onyinye Ukoha-kalu, B.Pharm, M.Pharm, Ph.D

Academic Editor

PLOS ONE

Dear Editor,

Thank you for considering our manuscript “Impact of Health System Interventions in Primary Health Settings on Type 2 diabetes care and Health Outcomes Among Adults in West Africa: A Systematic Review Protocol” for publication in your esteemed journal. We appreciate the time and effort devoted by the editors and reviewers to providing feedback on our work.

We have carefully considered the comments and revised the manuscript accordingly.

Our responses to the reviewer’s comments have been outlined below. The comments are in ***bold italic font*** and our responses are in regular font.

We believe these revisions have helped improve the quality and clarity of our manuscript significantly.

Your efforts in handling the manuscript are greatly appreciated.

Yours Sincerely,

Eugene Paa Kofi Bondzie.

(On behalf of all authors)

**REVIEWERS’ COMMENTS AND RESPONSES**

**Reviewer #3:**

**“*The use of ‘However’ in line 34 should be reconsidered in relation to the transition from the previous sentence.”***

Response:

Thank you for this comment. We have removed the word “However” from line 34.

***“Line 53 and 54, I don’t think this should be included in abstract but in main text.”***

Response

Thank you for your suggestion. We have removed lines 53 and 54 from the abstract. The limitations of the study have been maintained in the main text of the manuscript.

***“Line 49: This part of the sentence is not clear ‘and Car.info from inception to January 2024’***

Response

Thank you for your comment. The sentence has been re-worded as “We will also search Cairn.info for articles published between January 2005 to January 2024.”

***“Check for typos throughout, for example line 77/78 ‘These disproportionate number reflect the poor structures’ should be numbers.***

***Most of the information is there but there is repetitiveness and the paper could benefit from a language sweep overall to strengthen the presentation.***

Response

Thank you for your thoughtful suggestion. We have corrected all identified typos within the manuscript and removed repeated texts.
